# Re-Irradiation With Stereotactic Body Radiotherapy for In-Field Recurrence of Pancreatic Cancer After Prior Stereotactic Body Radiotherapy: Analysis of 24 Consecutive Cases

**DOI:** 10.3389/fonc.2021.729490

**Published:** 2021-11-02

**Authors:** Yuxin Shen, Xiaofei Zhu, Fei Cao, Hongliang Xie, Xiaoping Ju, Yangsen Cao, Shuiwang Qing, Zhen Jia, Lei Gu, Fang Fang, Huojun Zhang

**Affiliations:** Department of Radiation Oncology, Changhai Hospital Affiliated to Navy Medical University, Shanghai, China

**Keywords:** pancreatic cancer, re-irradiation, SBRT, in-field recurrence, toxicity

## Abstract

**Purpose/Objectives:**

Locally recurrent pancreatic cancer is a therapeutic challenge, and aggressive approaches are needed to improve its clinical outcomes. Stereotactic body radiotherapy (SBRT) is a promising treatment for pancreatic cancer with an excellent local control and acceptable toxicity. However, the safety and efficacy of SBRT for in-field recurrence after initial SBRT remain unknown. The aim of the study was to investigate the feasibility of re-irradiation with SBRT for locally recurrent pancreatic cancer after prior definitive SBRT.

**Material/Methods:**

Twenty-four consecutive patients with pancreatic cancer received two courses of SBRT in our center between January 2014 and December 2016. The median prescription dose of the initial and second courses of SBRT was 35.5 Gy/5–7f and 32 Gy/5–8f, respectively. Clinical outcomes including overall survival (OS), disease control, and toxicity were evaluated after treatment.

**Results:**

The median interval between two courses of SBRT was 13 months (range: 6–29 months). From the first SBRT, the median OS of 18 patients with limited diseases was 26 months (95% CI: 19.1–32.95 months). The median OS of 12 patients without metastasis was 14 months (95% CI: 10.6–17.4 months) from re-irradiation of SBRT. The overall response rate and disease control rate were 50% and 13%, and 100% and 86.9% after each SBRT, respectively. Carbohydrate antigen 19-9 (CA19-9) levels declined dramatically after re-irradiation within 1 month (p = 0.002) and 3 months (p = 0.028). Twelve (75%) out of 16 patients had pain relief after re-irradiation. None of the patients experienced gastrointestinal toxicity.

**Conclusions:**

Re-irradiation with SBRT can provide favorable outcomes and effective analgesia with mild toxicity after prior SBRT for in-field recurrent pancreatic cancer, which might be feasible for locally relapsed pancreatic cancer.

## 1 Introduction

Pancreatic cancer is one of the most lethal malignancies. The incidence and mortality have increased dramatically, and pancreatic cancer is projected to arise as the second leading cause of cancer-related deaths in Europe and the United States by 2030 (1–3). Stereotactic body radiotherapy (SBRT) is a promising treatment for pancreatic cancer with an excellent local control and acceptable toxicity (4). However, nearly one-third of patients treated with chemoradiation have local–regional recurrence (5), and 30% of deaths are due to locally progressive disease (6). Because of the symptomatic manifestations such as pain, obstruction, portal hypertension, and malnutrition, local progression is an important factor contributing to poor prognosis and deteriorations of quality of life (7, 8). The alternative aggressive approaches for local recurrence are warranted to improve the clinical outcomes.

Re-irradiation with SBRT has been successfully applied in lung cancer, head and neck cancer, bone metastases, and other cancers (9–12). It is a promising modality in the salvage setting due to its conformality and sparing dose to critical structures around pancreatic tumor, thus reducing the risk of significant late toxicity. The previous studies have proved that re-irradiation with SBRT might be an option for patients with locoregional recurrence after conventional fractionation radiotherapy (13–17). However, few studies have investigated the feasibility of two courses of SBRT for patients with locally relapsed pancreatic cancer. To the best of our knowledge, this pilot study was the one with a large sample size to investigate the safety and efficacy of re-irradiation with SBRT for in-field recurrence after initial SBRT for pancreatic cancer.

## 2 Materials and Methods

### 2.1 Study Population

This was a single-center retrospective study. Medical records of 24 consecutive patients treated with two courses of SBRT in our center from 2014 to 2016 were reviewed. Patients signed informed consent, and the study was approved by our institutional review board. The inclusion criteria included the following: i) patients with two courses of SBRT in our hospital, ii) in-field recurrences, iii) no involvement of the gastrointestinal tract by the tumor proven by imaging examinations and gastroscopy, iv) no upper gastrointestinal ulcer/active inflammation, and v) local recurrence confirmed by histopathological examinations or multidisciplinary approaches.

### 2.2 Stereotactic Body Radiotherapy Planning

The protocol of pancreatic SBRT has been described in our previous studies (18–20). SBRT was delivered by CyberKnife robotic stereotactic radiosurgery system (Accuray Inc., Sunnyvale, CA). Gross tumor volume (GTV) was delineated as a radiographically evident gross disease from both plain and contrast CT images. Clinical target volume (CTV) encompasses areas of the potential subclinical disease spread that equaled GTV. Planning target volume (PTV) was determined with 2- to 5-mm expansion from CTV. For tumors adjacent to critical organs, such as the duodenum, the expansion of PTV in this direction was reduced. Doses were prescribed to the 75%–80% isodose covering 95% of the GTV and 90% of the PTV. The radiation dose-fractionation schedule was based on the tumor size, tumor location, and the distance from organs at risk (OARs).

Regarding dose constraints of OARs, the American Association of Physicists in Medicine (AAPM) guidelines in TG-101 was referred (21). Additionally, in the case of re-irradiation with SBRT, a dose reduction of OARs was considered based on the previous study (19). Therefore, we allowed a dose reduction of 50% for a re-irradiation 12 months after the last radiation. A dose reduction of 25% was allowed for a re-irradiation 6–12 months after initial SBRT. No dose reduction was used when re-irradiation was performed within 6 months. Doses to OARs in the first SBRT would be modified according to the following conditions before summations of dose distributions of the first and second SBRTs. As a result, after completions of the treatment plans of re-irradiation, dose distributions, structures sets, and CT scans of two treatment plans were extracted from Multiplan^®^ System (version: 4.0.2) and sent to MIM^®^ System (version: 6.6.8) for analysis. Firstly, two CT scans were aligned rigidly *via* automatic bone matches (translation and rotations). Therefore, for each plan before summation, each of the contoured OARs was registered rigidly. Subsequently, a non-rigid registration was performed for projections of the dose distributions of the first plan to the second one for dose summations. The summed doses of OARs were compared with constraints. And the second plans would be modified if the summed doses exceeded the constraints. The dose constraints of OARs are listed in **Table 1**.

**Table 1:** Dose to critical organs in the first and second SBRTs.

OAR	Dose–volume limits	Median dose, Gy (range)		
		SBRT_1st^†^	SBRT_2nd^‡^	Sum^§^
Duodenum	D_max_	24.68	14.7	35.53
		(1.67–51.53)	(1.55–33.11)	(3.12–73.62)
	5 cm^3^	12.67	6.96	19.49
		(1.06–21.31)	(0.7–17.98)	(2.3–60.31)
Bowel	D_max_	30.31	18.43	44.36
		(17.51–40.98)	(10.89–35.49)	(29.7–92.47)
	5 cm^3^	19.18	13.17	29.92
		(11.35–28.38)	(6.46–25.71)	(16.89–43.45)
Stomach	D_max_	30.86	17.52	44.15
		(3.18–42.04)	(3.01–39.7)	(7.64–44.15)
	10 cm^3^	16.43	9.35	26.74
		(2.11–25.11)	(1.22–21.05)	(3.49–32.63)
Spinal cord	D_max_	5.62	2.92	8.54
		(2.43–11.64)	(1.04–16.92)	(4.44–18.88)
	0.35 cm^3^	5.21	2.67	7.93
		(2.18–10.15)	(0.93–13.64)	(3.99–14.93)
Liver	D_mean_	2.82	1.7	5.29
		(0.76–8.72)	(0.83–5.63)	(1.96–10.7)
Left kidney	<2/3	1.74	1.14	3.2
		(0.55–6.64)	(0.62–3.4)	(1.61–7.96)
Right kidney	<2/3	1.44	0.88	2.56
		(0.7–3.48)	(0.6–3.13)	(1.49–4.93)

Note. SBRT, stereotactic body radiotherapy; OAR, organ at risk.

^†^SBRT_1st: the first course of SBRT.

^‡^SBRT_2nd: the second course of SBRT.

^§^Sum: the dose summation of two plans using non-rigid registration by MIM^®^System.

### 2.3 Follow-Up

The primary endpoints included overall survival (OS) and late grade 3 and higher radiation toxicity. Adverse effects induced by SBRT were evaluated by Common Terminology Criteria for Adverse Events (CTCAE) Version 4.03. The toxicity was evaluated every month. The secondary endpoints included local recurrence-free survival (LRFS), overall response rate (ORR), disease control rate (DCR), and numerical pain rating scale (NPRS). The OS was defined from the initial date of re-irradiation to death. The LRFS was determined from the initial date of re-irradiation to local recurrence or death. The ORR is a ratio of the number of patients with complete response (CR) plus partial response (PR) to the total number of patients eligible for evaluations. The DCR is a ratio of the number of CR, PR, plus stable disease (SD) to the total number of patients included for analysis. The definition of objective response was referred to Response Evaluation Criteria in Solid Tumors version 1.1 (RECIST v1.1). In-field recurrence was defined as more than 80% of the recurrence volume located in the prescription dose line.

### 2.4 Statistical Analysis

Statistical analysis was performed with SPSS 20.0 (IBM Corporation, Armonk, NY, USA). Survival was assessed by Kaplan–Meier plots. The tests for significance were performed by the log-rank method. The difference between groups was analyzed by Wilcoxon rank sum test. A swimmer’s plot was created to depict individual disease timelines.

## 3 Results

### 3.1 Patients

**Figure 1:**
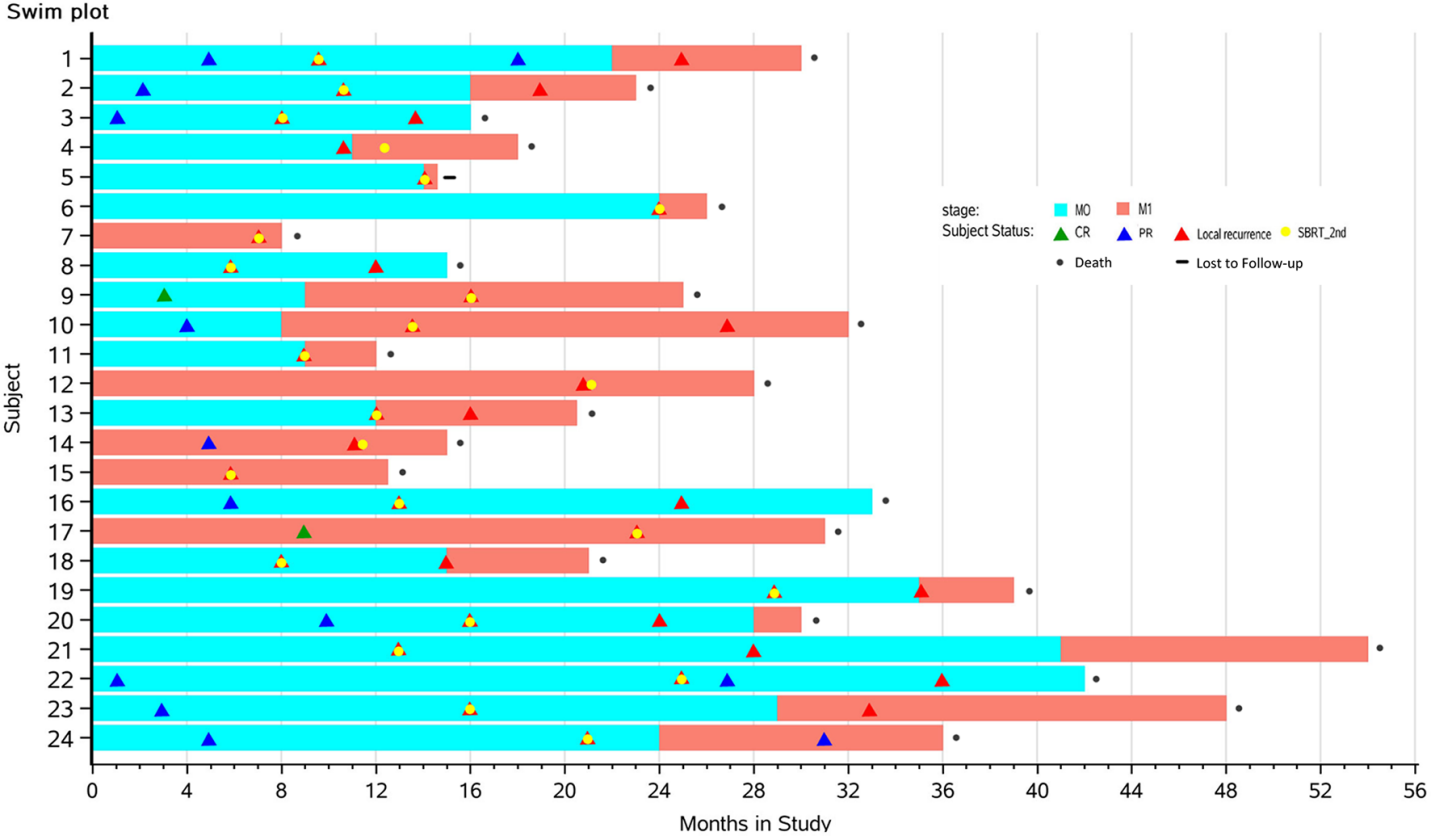
Swimmer plots depicting time course of pancreatic cancer from the first SBRT to death in 24 patients with complete follow-up data. Yellow dots mark the times that the patients received the second SBRT. Blue blocks represent stages with limited diseases (M0), while red portions represent distant metastasis (M1). Triangles mark times when CR (green), PR (blue), or local recurrence (red) was diagnosed by imaging. SBRT, stereotactic body radiotherapy; CR, complete response; PR, partial response.

Between January 2014 and December 2016, 24 consecutive patients were included. Patients’ characteristics are summarized in **Table 2**, and individual disease courses were depicted in a swimmer’s plot (**Figure 1**). Median follow-up from re-irradiation was 9 months (range, 1–41 months); and one (3.8%, No. 6) patient was lost to follow-up. Median age at the time of re-irradiation was 67 years (range, 43–84 years). The majority of patients had stage T4 disease located in the head of pancreas. Five of 24 patients (liver, Nos. 7, 14, 15, 17; and lung, No. 12) already had metastasis at initial SBRT, while another seven (liver, Nos. 4–6, 9, and 13; lung, No. 10; and peritoneum, No. 11) developed metastases along with local recurrence after initial SBRT. At time of repeat SBRT, 50% had only local disease and 50% had both local and distant metastases.

**Table 2:** Patients characteristics.

Total no. of patients enrolled	24	
Gender, n		
Male	16	
Female	8	
Median age, years (range)	65.5 (39–83)	
Location, n		
Head	12	
Neck	3	
Body	9	
	SBRT_1st^†^	SBRT_2nd^‡^
Primary tumors		
T1c	2	2
T2	7	7
T3	3	3
T4	12	12
Regional lymph nodes		
N0	9	9
N1	11	11
N2	4	4
Metastasis		
M0	19	12
M1	5	12
Stage		
I	IA: 2; IB: 2	IA: 2
II	IIA: 1; IIB: 1	IIB: 1
III	13	9
IV	5	12
KPS		
90	6	10
80	18	14

Note. SBRT, stereotactic body radiotherapy; KPS, Karnofsky Performance Status.

^†^SBRT_1st: the first course of SBRT;

^‡^SBRT_2nd: the second course of SBRT.

### 3.2 Treatment Regimens

#### 3.2.1 Stereotactic Body Radiotherapy Planning and Delivery Variables

The median time between the first and second SBRTs was 13 months (range, 6–29 months). At the time of the first SBRT, the median volume of PTV was 34.8 cm^3^ (range, 14.8–196.1 cm^3^), and the median maximum diameter of GTV was 31 mm (range, 14–46 mm). The median prescription dose and biologically effective dose (BED) was 35.5 Gy/5–7f (range, 32–42 Gy) and 60.7 Gy (range, 52.5–72 Gy), respectively. Most patients (22/24) underwent 5-fraction schedule (median: 35.5 Gy, range: 32–40 Gy). The other two received 42 Gy/6f (No. 20) and 35 Gy/7f (No. 24), respectively. The median volume of PTV of re-irradiation was 18.7 cm^3^ (range, 10.4–139.5 cm^3^), and the median maximum diameter of GTV was 28 mm (range, 13–44 mm). The median prescription dose and BED of re-irradiation were 32 Gy/5–8f (range, 25–40 Gy) and 51.4 Gy (range, 37.5–72 Gy), respectively. Sixteen patients (68%) had 5 fractions (median: 31.5 Gy, range: 25–40 Gy). Four (Nos. 12, 13 18, and 20) patients received 6 fractions (median: 35.4 Gy, range: 31.8–36 Gy); and three (Nos. 17, 22, and 24) received 8 fractions (median: 29.6 Gy, range: 29–32 Gy). One (No. 23) received 35 Gy/7f.

#### 3.2.2 Chemotherapy

Half of the patients received gemcitabine or 5-FU-based chemotherapy regimens for at least 6 cycles after the first SBRT. Chemotherapy was interrupted in five patients (20.1%; Nos. 3, 4, 9, 15, and 24) due to adverse effects. The remaining seven patients (29.2%; Nos. 6–8, 11, 12, 21, and 23) declined chemotherapy because of older age or morbidity. After re-irradiation, due to the poor physical condition or severe clinical manifestations of pancreatic cancer, 14 patients (58.3%) did not receive the chemotherapy. Only three patients (12.5%) completed six cycles of chemotherapy (doublet chemotherapy, No. 2, 10; 5-FU alone, No. 16). Seven patients (Nos. 3, 13, 15, 18–20, and 22) could not tolerate and interrupt chemotherapy.

#### 3.2.3 Surgery

Four patients received surgical resections before the first SBRT. One (No. 10) of them had local recurrence at the anastomotic stoma 9 months after distal pancreatectomy. Three (Nos. 17, 20, and 23) had metastasis of regional lymph nodes at the median time of 12 months (range, 9–24 months) after pancreatoduodenectomy. One patient (No. 3) with stage III (T4N0M0) underwent palliative surgery 4 months after re-irradiation because of the rise of carbohydrate antigen 19-9 (CA19-9), while the response was SD. Unfortunately, the patient suffered from seeding metastasis in the peritoneal cavity within 2 months and died 5 months after the operation.

### 3.3 Prognosis

**Figure 2:**
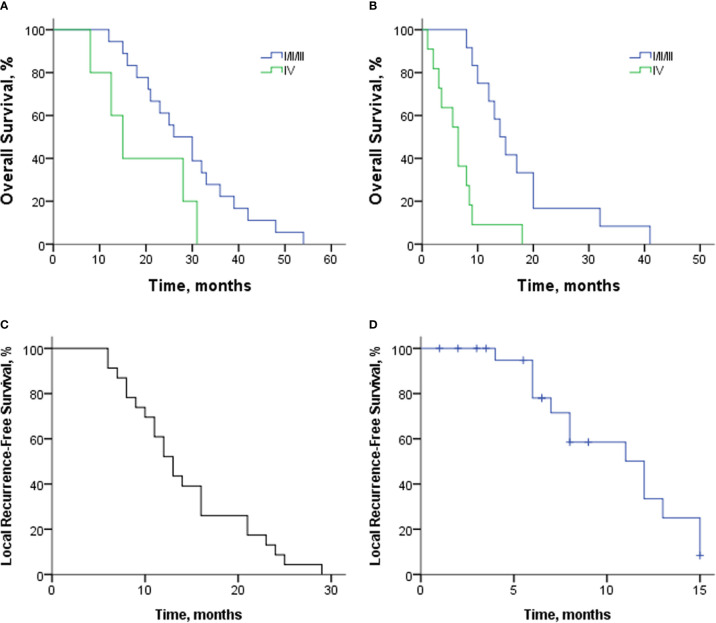
**(A)** Overall survival of patients with or without distant metastasis from the first SBRT. **(B)** Overall survival of patients with various stages from re-irradiation with SBRT. **(C)** LRFS of 23 patients from the first SBRT. **(D)** LRFS of 23 patients from re-irradiation with SBRT. SBRT, stereotactic body radiotherapy; LRFS, local recurrence-free survival.

Twenty-three patients were included for survival analysis. The OS of patients with or without distant metastasis from the first SBRT is shown in **Figure 2A**. The median OS of 18 patients with limited diseases (M0) was 26 months (95% CI: 19.1–32.95 months). The 1-, 2-, and 3-year OS rates were 94.4%, 61.1%, and 22.2%, respectively. The median OS of five patients with distant metastasis was 15 months (95% CI: 9.6–20.4 months).

From the time of re-irradiation, the median OS of patients with limited diseases and distant metastasis was 14 months (95% CI: 10.6–17.4 months) and 6.5 months (95% CI: 3.4–9.6 months); **Figure 2B**. The 12-, 18-, and 24-month OS rates of 12 patients with limited diseases were 66.7%, 33.3%, and 16.7%, respectively. At the time of re-irradiation, there were two patients with stage Ia. One was a 77-year-old female patient (No. 21) who did not receive chemotherapy and survived 41 months after re-irradiation. Another patient (No. 2), male, 56 years old, and could not tolerate surgery due to severe emphysema, received 13 and 6 cycles of doublet chemotherapy after the first and second courses of SBRT. Four months after re-irradiation, the tumor response was SD, but the CA19-9 level increased (from 23.19 to 43.14 U/ml). However, the patient himself had a strong desire to receive aggressive treatment and then received Gamma Knife at another center. Unfortunately, he hemorrhaged 7 months after radiotherapy and died of gastrointestinal fistula.

The median LRFS from the first SBRT was 13 months (95% CI: 10.7–15.3 months; **Figure 2C**). The rate of 6-, 12-, and 18-month LRFS was 91.3%, 52.2%, and 26.1%, respectively. After re-irradiation with SBRT, 13 patients had local progression, and 10 patients died without local progression. The median LRFS from re-irradiation was 12 months (95% CI: 7.9–16.1 months; **Figure 2D**). The 6- and 12-month LRFS rates were 78% and 33.4%, respectively.

### 3.4 Objective Response

The best tumor response of each patient was marked in swimmer plots (**Figure 1**) with differently colored triangles (details provided in Subject Status).

**Figure 3:**
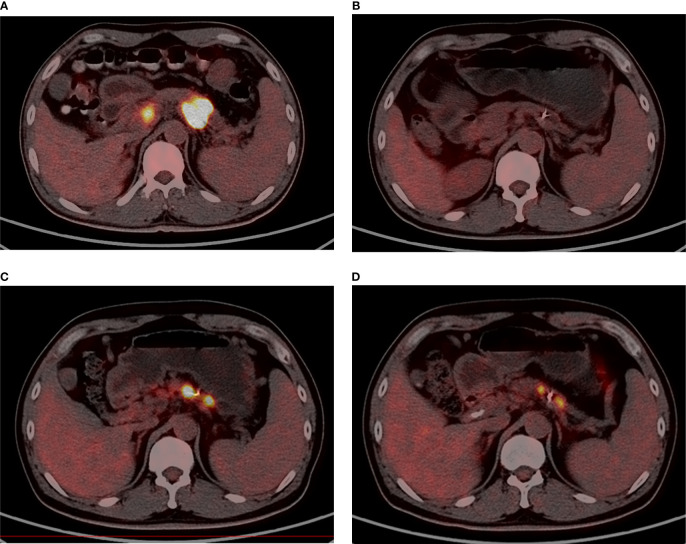
A male, 47 years old, who has adenocarcinoma, with stage III before the first SBRT [**(A)**; SUVmax = 23.2]. Nine months after the first SBRT (35 Gy/7Fx), the tumor had shrunk drastically [**(B)**; SUVmax = 8.0]. The patient relapsed at 22 months after the first SBRT and underwent re-irradiation [**(C)**; SUVmax = 21.8]. Four months after re-irradiation (32 Gy/8Fx), the SUV value declined [**(D)**; SUVmax = 9.2].

After the first course of SBRT, CR was found in two patients at the time of months 3 and 9. Ten patients had PR, and 12 had SD. The ORR was 50%, and the DCR was 100% after the first SBRT. After re-irradiation, three patients had PR, and 17 patients had SD marked as the best tumor response. Three patients with distant metastasis died before radiographic examinations. The ORR was 13%, and the DCR was 86.9% after re-irradiation. **Figure 3** shows one patient’s image (No. 24) with ^18^F-FDG PET/CT during the two courses of SBRT.

### 3.5 Evaluation of Carbohydrate Antigen 19-9 and Pain

**Figure 4:**
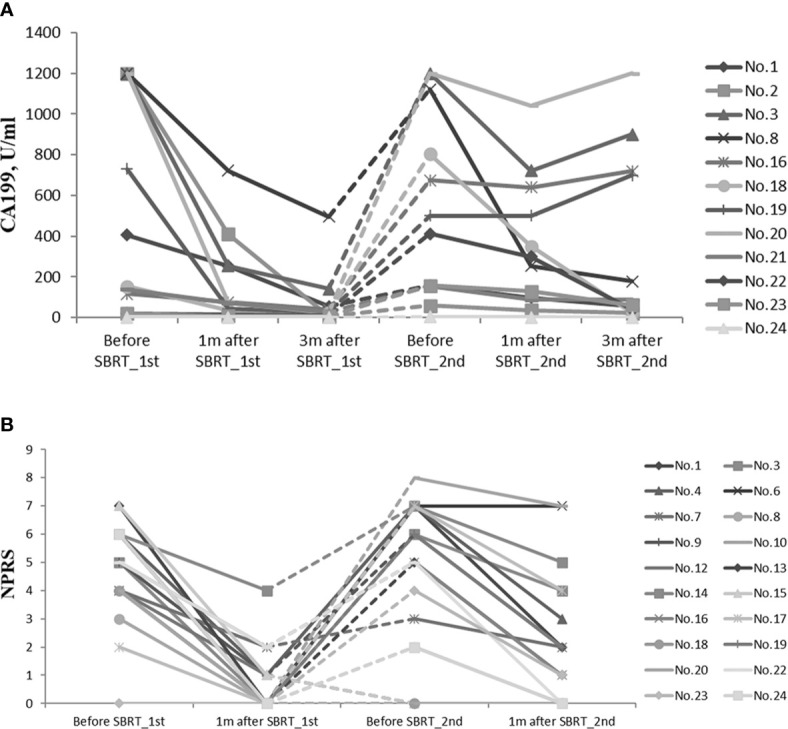
**(A)** CA19-9 response of 13 patients with limited diseases during the whole treatment. **(B)** NPRS assessment of 22 patients during the whole treatment. CA19-9, carbohydrate antigen 19-9; NPRS, numerical pain rating scale.

Considering the impact of metastasis on CA19-9, 12 patients with limited diseases at re-irradiation were included to assess CA19-9 response (**Figure 4A**). Compared with baseline before the first SBRT, CA19-9 level declined dramatically in 1 month (p = 0.002). Three months later, the level was lower than that at 1 month after the first SBRT (p = 0.004). After re-irradiation of SBRT, the CA19-9 level decreased significantly within 1 month (p = 0.003) and 3 months (p = 0.041).

In both courses of SBRT, cancer-induced pain was ameliorated (**Figure 4B**). Before the first SBRT, the median NPRS of 19 patients was 5 (range, 2–7). One month after the first SBRT, the score declined rapidly with the median score of 0 (range, 0–4, p = 0.000). Eighteen patients (94.7%) experienced alleviation of the pain during the treatment, while 11 patients (57.9%) had complete remission at the time of completion of SBRT. Before re-irradiation of SBRT, 16 patients suffered from epigastric pain, and the median NPRS was 6 (range, 2–8). One month later, the median score decreased to 2 (range, 0–7, p = 0.000). The pain was relieved in 12 patients (75%) during the treatment, while nine patients (56.3%) reported significant remission after the second SBRT.

### 3.6 Toxicity

Twenty-two patients were included for evaluation of toxicity except for one patient who received Gamma Knife in another center for the same lesion after re-irradiation.

No patients experienced grade 4 or 5 toxicity. No one suffered from late gastrointestinal toxicity. One patient developed acute grade 3 vomiting and diarrhea after re-irradiation and recovered after hospitalization. Grade 2 nausea and emesis were found in one patient after the first SBRT. Additionally, five patients had acute grade 2 toxicity after re-irradiation (nausea, 3; vomiting, 1; anergy, 2; diarrhea, 2; myelosuppression, 2).

## 4 Discussion

Given local progression after initial aggressive treatment, management of pancreatic cancer becomes more challenging. There were limited treatment options for patients with locally relapsed pancreatic cancer after aggressive multimodality, and no consensus has been reached on the optimal treatment strategy. These patients are usually non-surgical candidates due to factors such as age, comorbidities, performance status, or advanced tumor stage with vascular involvement or post-radiation fibrosis. In studies evaluating different alternatives for isolated local recurrence of pancreatic cancer after various regimens, median survival was reported with 32, 19, and 16 months for re-resection, chemoradiotherapy, and SBRT, respectively (22). However, because patients with different stages were included, the optimal treatment still remained controversial. Each of these options has its own drawbacks, for instance, the invasiveness and morbidity with re-resection; poor response rates, systemic toxicity, and modest local control with palliative chemotherapy (23, 24); significant morbidity such as pain and obstruction with radiotherapy (25); and lack of efficacy with supportive care alone. It is believed that these patients are most likely to benefit from intensive local therapy.

SBRT provides a high local control and potential survival benefits for pancreatic cancer. Till now, only five retrospective studies reported prognosis and toxicity of SBRT as treatment options for patients with recurrent pancreatic cancer who had received prior radiotherapy (including conventional fractionation radiotherapy and SBRT). In **Table 3**, we have summarized the published five studies with different focus. Conventional fractionation radiotherapy was commonly applied as the initial treatment. Only 10 patients reported by Dagoglu et al. (15) received two courses of SBRT, whereas detailed patients’ characteristics and outcomes were not disclosed. The median OS of patients with limited diseases from re-irradiation in our study was 14 months and was comparable with that reported by Dagoglu et al. However, half of the included patients in the study had stage I and II, while 75% (9/12) of patients in our study had stage III. In the remaining four published studies (13, 14, 16, 17), the median OS with limited diseases was 6–9 months after re-irradiation with SBRT. Hence, our study showed superior survival rate compared with that in previous studies, which might be attributable to the two courses of SBRT and higher prescription dose for these patients.

**Table 3:** Studies published until 2018 evaluating the impact of SBRT re-irradiation on local pancreatic cancer recurrence vis-à-vis the current study.

Study	Patients, (n)	Stage (n)	Initial radiotherapy	Median dose of re-irradiation	Median OS (months)	Toxicity
Lominska et al. (13), 2012	28	–	CFRT^†^	22.5Gy/3-5f^‡^: Boost SBRT (11) and salvage SBRT (17)	5.9	14.3% grade 3 (late): bowel obstruction (1) and gastric perforation (1)
Wild et al. (14), 2013	18	–	CFRT	25Gy/5f	8.8	6% grade 3 (late): bowel obstruction (1)
Dagoglu et al. (15), 2016	30	I/II: 14; III: 16	CFRT (15); SBRT (10); salvage CFRT (5)	25Gy/5f	14	7% grade 3 (late): bowel obstruction (2)
Koong et al. (16), 2017	23	N0: 15; N1: 8	CFRT	25 Gy/1f (9) 25 Gy/5f (14)	8.5	8.7% grade 3 (acute): gastric hemorrhage (1) and gastric fistula (1)
Sutera et al. (17), 2018	38	–	CFRT	24.5 Gy/1–3f	9.7	7.9% grade 3 (late): nausea (2) and enteritis (1) 2.6% grade 4 (late): duodenal stenosis (1)
Current study, 2021	22	IA: 2; IIB: 1; III: 9; IV: 12	SBRT	32 Gy/5–8f	M0: 14; M1: 6.5	no grade 3 (late); 4.5% grade 3 (acute): vomiting and diarrhea (1)

Note. SBRT, stereotactic body radiotherapy; OS, overall survival.

^†^CFRT, conventional fractionation radiotherapy.

^‡^Fx, fraction.

The median prescription dose and fractionation adopted in our study were higher than in the previous studies (13–16), which might be associated with a satisfactory local control rate and a very low toxicity profile. No patients had late adverse events after re-irradiation in this study, and the incidence was much lower than that in the previous investigations (13–15, 17). None of the patients experienced small intestine obstruction and duodenal stenosis. This might be attributed to the use of SBRT as initial treatment and the 5–8 fractions used in re-irradiation. Koong et al. (16) reported that 26.1% of patients developed grade 2 or 3 gastrointestinal toxicity, two-thirds of whom received a single-fraction SBRT regimen. Lominska et al. (13) and Sutera et al. (17) showed that the late gastrointestinal toxicity above grade 3 often occurred in patients who underwent 1–3 fractions. Therefore, it was suggested that a multi-fraction SBRT regimen may result in less toxicity. The fractionation schedule in our study comprised of 5–8 fractions, which might contribute to the better tolerance. Koong et al. (16) have specified for the adequate time for allowing normal tissues to repair before the delivery of a new course of radiation treatment, particularly when using hypofractionation. This was in line with our study with the median interval of 13 months (range, 6–29 months) between the first and second SBRTs, which led to the low incidence of adverse effects in our study.

CA19-9 is considered as one of the most useful biomarkers for the assessment of pancreatic cancer (26). Our previous studies (20) developed a predictive model for stratification of patients with pancreatic cancer who may achieve survival benefits from re-irradiation with SBRT. Multivariable analysis showed that tumor stage, BED_10_, and CA19-9 response were significantly predictive of OS, which formed the components of Stage, CA19-9 response, BED_10_ (SCAD) scoring system. Our study was the only one evaluating CA19-9 levels in this setting. It was observed that CA19-9 levels declined significantly within 1 month (p = 0.002) and 3 months (p = 0.028), compared with those prior re-irradiation with SBRT.

Approximately 70% of patients suffered pain at the time of diagnosis (27). The significant pain relief was observed soon after SBRT in most patients, including patients with distant metastasis. There was a 4-point reduction in median NPRS after re-irradiation of SBRT in 16 patients who experienced epigastric pain. Similarly, pain was also alleviated in 75% of patients during the treatment, and 56.3% of patients reported significant remission after the second SBRT. Hence, we inferred that SBRT was a better option for analgesia.

In terms of RECIST response rates, the percentage of ORR and DCR was 50% and 100% after the first SBRT and 13% and 86.9% after re-irradiation, which may indicate that the purpose of re-irradiation was disease and pain control. However, three patients with distant metastasis died within 3 months after re-irradiation. Therefore, although re-irradiation with SBRT may contribute to effective analgesia for patients with metastatic pancreatic cancer, careful pretreatment evaluations were required for selection of patients who were indicated for re-irradiation with SBRT.

The results of this study should be interpreted within the context of its limitations, including being a single-center study, having a retrospective nature, non-randomization, and a small sample size. In spite of the limitations, the study had strengths that should be acknowledged. To our knowledge, this is the first study that integrated the pain parameters and CA19-9, which need to be correlated with other investigations for better clinical judgement.

## 5 Conclusions

Re-irradiation with SBRT is a feasible strategy for in-field recurrence of pancreatic cancer after prior SBRT, with favorable OS rate and acceptable treatment-related toxicity, especially in patients with no distant metastases. Pain remission should be considered for selected patients with metastatic pancreatic cancer if a second SBRT was performed. Two courses of SBRT, a higher dose of re-irradiation, and multi-fraction schemes might result in favorable local control and survival with acceptable toxicity.

## Data Availability Statement

The original contributions presented in the study are included in the article/supplementary material. Further inquiries can be directed to the corresponding author.

## Ethics Statement

Written informed consent was obtained from the individual(s) for the publication of any potentially identifiable images or data included in this article.

## Author Contributions

YS, XZ, FC, and HX designed the experiments and wrote the paper. YC helped to analyze the treatment planning. HZ revised the paper. All authors contributed to the article and approved the submitted version.

## Funding

This study was sponsored by grants from the Special Project of Ministry of Science and Technology (2017YFC0113104) and the National Natural Science Foundation of China (Grant No. 81803168).

## Conflict of Interest

The authors declare that the research was conducted in the absence of any commercial or financial relationships that could be construed as a potential conflict of interest.

## Publisher’s Note

All claims expressed in this article are solely those of the authors and do not necessarily represent those of their affiliated organizations, or those of the publisher, the editors and the reviewers. Any product that may be evaluated in this article, or claim that may be made by its manufacturer, is not guaranteed or endorsed by the publisher.
